# Emotionality vs. Other Biobehavioural Traits: A Look at Neurochemical Biomarkers for Their Differentiation

**DOI:** 10.3389/fpsyg.2021.781631

**Published:** 2021-12-20

**Authors:** Irina N. Trofimova, Anastasia A. Gaykalova

**Affiliations:** ^1^Laboratory of Collective Intelligence, Department of Psychiatry and Behavioural Neurosciences, McMaster University, Hamilton, ON, Canada; ^2^Department of Psychology, McMaster University, Hamilton, ON, Canada

**Keywords:** dispositional emotionality, FET model, opioid receptor, hormones, empathy, sensation seeking, neurochemical biomarkers

## Abstract

This review highlights the differential contributions of multiple neurochemical systems to temperament traits related and those that are unrelated to emotionality, even though these systems have a significant overlap. The difference in neurochemical biomarkers of these traits is analysed from the perspective of the neurochemical model, Functional Ensemble of Temperament (FET) that uses multi-marker and constructivism principles. Special attention is given to a differential contribution of hypothalamic–pituitary hormones and opioid neuropeptides implicated in both emotional and non-emotional regulation. The review highlights the role of the mu-opioid receptor system in dispositional emotional valence and the role of the kappa-opioid system in dispositional perceptual and behavioural alertness. These opioid receptor (OR) systems, microbiota and cytokines are produced in three neuroanatomically distinct complexes in the brain and the body, which all together integrate dispositional emotionality. In contrast, hormones could be seen as neurochemical biomarkers of non-emotional aspects of behavioural regulation related to the construction of behaviour in fast-changing and current situations. As examples of the role of hormones, the review summarised their contribution to temperament traits of Sensation Seeking (SS) and Empathy (EMP), which FET considers as non-emotionality traits related to behavioural orientation. SS is presented here as based on (higher) testosterone (fluctuating), adrenaline and (low) cortisol systems, and EMP, as based on (higher) oxytocin, reciprocally coupled with vasopressin and (lower) testosterone. Due to the involvement of gonadal hormones, there are sex and age differences in these traits that could be explained by evolutionary theory. There are, therefore, specific neurochemical biomarkers differentiating (OR-based) dispositional emotionality and (hormones-based) body’s regulation in fast-changing events. Here we propose to consider dispositional emotionality associated with OR systems as emotionality in a true sense, whereas to consider hormonal ensembles regulating SS and EMP as systems of behavioural orientation and not emotionality.

## Introduction: Functional Constructivism in Sorting Neurochemical Biomarkers of Temperament Traits and Psychopathology

### A Neurochemical Mix-up Between Systems Implicated in Emotionality and Non-emotional Regulation

The further that research in the neurochemistry of emotionality advances, the more that neurochemical systems are being linked to emotional regulation. In fact, a dysregulation in practically all neurochemical families – monoamines (MAs), acetylcholine (ACh), GABA, hormones, neuropeptides, opioid receptors (ORs), transcription factors, etc. – appears to contribute to mood and anxiety disorders. However, the same neurochemical systems were implicated in the regulation of non-emotionality aspects of behaviour (attention, planning, habits, plasticity, and, as discussed below – empathy). There is, therefore, a functional overlap of neurochemical systems regulating aspects of behaviour related and unrelated to emotionality. As discussed elsewhere (Trofimova et al., 2018; Trofimova, 2021a,b; Trofimova and Netter, 2021), sorting through the neurochemical biomarkers of specific aspects of behavioural regulation is a very challenging task, requiring not only experimental research and statistical models but also detailed meta-analysis. The challenges arise from the fact that neurochemical systems regulate each other, have multiple functionalities, and represent multiple neurotransmitters and mediators. Each of these neurotransmitter systems has several types of receptors, each having different functionality and locations in the brain. Since all of these systems are contingent on each other’s activities, a one-to-one mapping between neurochemical systems and specific functional aspects of behaviour, temperament traits or symptoms of psychiatric disorders would be, therefore, not a very efficient way to present their interplay. Instead, an ensemble (multi-marker) presentation might be more fruitful.

In sorting out neurochemical systems of emotionality, one more complicating factor is that *emotionality*, despite the old age of this concept, is still poorly defined and often defined circularly (for example, “emotionality is how people express and feel their emotions”). There is no consensus or clear definition of emotionality even among psychologists. The American Psychological Association (APA) defines emotionality mostly based on its arousal component, as “the degree to which an individual experiences and expresses emotions, irrespective of the quality of the emotional experience.” Yet, there is another, a well-documented component of emotionality related to emotional valence (i.e., emotions having either positive, secure-to-happy or negative, dysphoric types) (Barrett and Bliss-Moreau, 2009; Barrett, 2017). In fact, without emotional valence (i.e., not having negative or positive affectivity), a solo component of arousal would not be experienced as emotion. This is what happened in classic experiments in the 1920–1940s when participants were asked to report their feelings after they were injected with epinephrine (adrenalin) (reviewed in Dror, 2016). Although the majority of participants reported feeling aroused, only a few experienced something emotional. Those who did report an emotional experience described it as having “cold,” and not genuine emotion. For example, in fearful contexts, they reported feeling “as if I am afraid” rather than being really afraid. The role of epinephrine and other hormones in behavioural arousal justified Schashter proposal to include arousal to components of emotionality, in addition to the cognitive appraisal component (Schachter and Singer, 1962).

One of the approaches that we found beneficial in sorting through neurochemical biomarkers of consistent behavioural patterns (CBPs) (temperament traits and symptoms of psychopathology) is the functional constructivism (FC) approach.

### Functional Constructivism Approach

The *Constructivism* approach has been known for about a 100 years in general psychology (Bartlett, 2009; see for review Trofimova, 2017, 2021a,b) and especially in the psychophysiology of emotions (Russell, 2003; Vuilleumier, 2005; Ghashghaei et al., 2007; Barrett, 2009, 2017; Pessoa, 2010). This approach suggests that all behaviour is not reactive but constructive; all actions are constructed anew as the integration of behavioural capacities, needs, and alternatives that were selected and sequenced for their relevance across multiple levels of behavioural regulation. Even when consistent behaviour looks like cycles, habits, and repetitions, it is actually never repeated but generated anew based on an individual’s current capacities, intentions, and situational context (Bernstein et al., 1996). A descendent of Constructivism is the FC approach that classifies the functions of neurobiological systems by their contribution to action construction. Neurobiological systems can have multiple and overlapping functionalities, and it is our position that this functionality was reinforced in evolution by its role in the sustainability of both individual and species functioning. To classify the functionality of human neurotransmitter systems, the FC suggests, therefore, looking at the stages and functional aspects of action construction identified in the sciences of kinesiology and functional neurophysiology that specifically analyse these stages. The pioneers of this analysis were Bernstein, who is credited as the father of kinesiology (Bernstein et al., 1996), and Anokhin (Anokhin and Serzhantov, 1973; Anokhin, 1984), the author of the functional systems theory.

**FIGURE 1:**
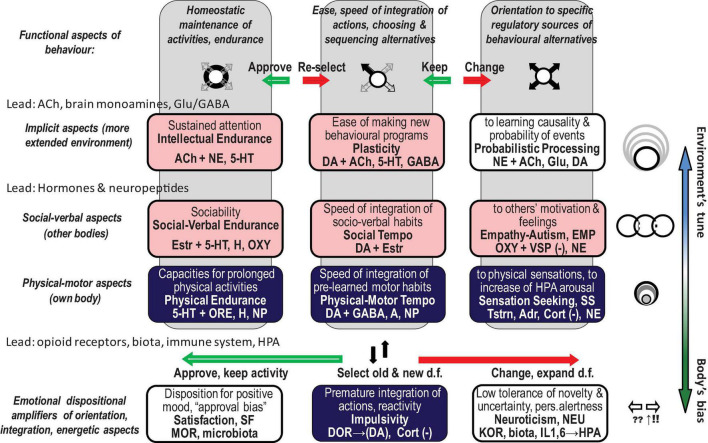
Twelve components of the neurochemical model Functional Ensemble of Temperament (FET) and functional aspects of action construction that these components regulate (bold italic font). The components are named as temperament traits (in bold font). Green shallow arrows show the directionality of contributions from mu, and red solid arrows – from kappa, opioid receptors. The components with light (pink) shadow are more expressed in individuals with high estrogen (like in many females), whereas components coloured in dark (blue) are more expressed in individuals with higher testosterone (like in many males). This influence of gonadal hormones is in line with predictions of the Evolutionary Theory of Sex. 5-HT, 5-hydroxytryptamine (serotonin); ACh, acetylcholine; NE, noradrenaline; DA, dopamine; NP, some hypothalamic neuropeptides and hormones; Glu, glutamate; GG, glutamate and GABA; H, histamine; A, adenosine; ORE, orexins; KOR, MOR, and DOR, kappa-, mu-, and delta-opioid receptors correspondingly. HPA, hypothalamic–pituitary–adrenal axis; GC, glucocorticoids; OXT, oxytocin; VSP, vasopressin; Estr, estrogen; Tstr, testosterone; Adr, adrenaline; Cort, cortisol. (–), contributed to the trait when low; d.f., degrees of freedom in action.

The FC approach to bio-behavioural taxonomies was implemented in the neurochemical framework Functional Ensemble of Temperament (FET) that summarised the most conservative findings regarding the functionality of neurotransmitters, hormones, and OR systems (Trofimova, 2016, 2018, 2021a,b; Trofimova and Robbins, 2016). The temperament of healthy individuals (including animals) and symptoms of psychiatric disorders can be presented as a continuum of weak vs. strong dysregulation within neurochemical systems generating CBPs (Sulis, 2018; Trofimova and Sulis, 2018; Rezaei et al., 2021; Trofimova, 2021b). This conception of the continuum is supported by decades of research in psychopharmacology and neurochemistry, showing the impact of neurochemical dysregulation (Sulis, 2018). The FET uses a “*multimarker*” approach, suggesting that there is no one-to-one correspondence between a specific CBP and any neurochemical system. Instead, every psychiatric symptom or temperamental (bio-behavioural) trait is associated with a team of neurochemical systems (Figure 1; Trofimova, 2016, 2018, 2021a,b; Trofimova and Robbins, 2016).

People face a vast number of behavioural alternatives, and so they must select which is most appropriate to a given situation for behaviour to be organised. Before this selection can be completed, however, individuals have to consider and reject multiple alternatives (degrees of freedom, d.f.) that are less suitable. This expansive consideration of alternatives is called here *behavioural orientation*. Selection of the most suitable d.f., sequencing them and suppressing the remaining d.f. is called *integration, or programming*. Generating similar actions using chosen integrations is called *maintenance* of behaviour. The FET framework follows Luria’s insights from clinical neuropsychology and insights from kinesiology, which identify three functional aspects of action construction: expansion of d.f. (orientation), selection/integration of a programme of action, and energetic maintenance (including decomposition of unneeded d.f.) (three columns of Figure 1). The FET also follows the *activity-specific approach* to taxonomies of temperament that differentiates between biomarkers regulating physical, verbal–social, and mental aspects of behaviour (Trofimova and Sulis, 2011; Trofimova, 2016; Trofimova and Robbins, 2016; Rusalov, 2018) (three top rows of Figure 1). The validity of this approach is supported neuroanatomically (by the functional specificity of brain areas regulating physical, verbal, and probabilistic aspects of behaviour), neurochemically (by the specificity of hypothalamic hormones differentially regulating social and physical aspects of behaviour) (Vazquez-Borrego et al., 2018; Swaab et al., 2021), and clinically (by differentiating between major psychiatric diagnoses) (Trofimova and Christiansen, 2016; Trofimova and Sulis, 2016a,b, 2018).

Moreover, analysis of neurochemical biomarkers of universal functional aspects of behavioural regulation showed that in terms of timing, they can be separated to those that regulate “here and now,” fast, explicit changes in actions and those that relate to eventual (“ever”), implicit and probabilistic aspects of events (Trofimova, 2021a). Our review focuses on this distinction, further analysing the roles of hormones in fast-changing situations and the roles of ORs in dispositional emotionality.

## The Roles of Opioid Receptor Systems in Emotional Valence and Alertness

### The Role of Endorphins Binding to Mu-Opioid Receptors in Dispositional Emotional Valence

Due to space limitations, we will mention the role of OR systems only briefly since this role is discussed in greater detail in other reviews (Leknes, 2008; Trofimova, 2016, 2018, 2021a,b; Trofimova and Robbins, 2016; Bodnar, 2018).

Opioid receptor systems are well-documented players in emotionality (Bodnar, 2018). Moreover, more and more evidence is emerging suggesting the role of gut microbiota and the immune system in the regulation of dispositional emotionality (Cryan and Dinan, 2012; Fung et al., 2017). OR action is differentially complemented or contra-influenced by the action of microbiota and cytokines of the immune system. Therefore, whenever there is a reference to the action of the OR system, we refer to the action of OR, microbiota and immune systems as one complex. In contrast to microbiota, which rarely has direct access to brain structures, influencing them indirectly, the opioid peptides have a more direct way to regulate brain systems. We appreciate that these three systems (OR, microbiota, and immune cells) are entirely different neurochemically and biologically, ranging from peptides to complex cellular organisms. Thus combining them into one “body-biased” system is, therefore an over simplification and is done here temporarily for conceptual purposes.

**FIGURE 2:**
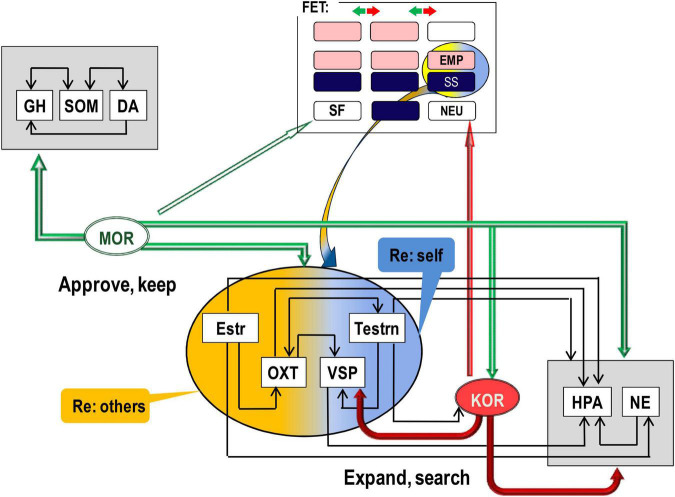
Differential contributions of (mu, MOR; kappa, KOR) opioid receptor systems and hormones to behavioural regulation. In line with predictions of the Evolutional Theory of Sex, interaction of estrogen (Estr, more expressed in females) with oxytocyn (OXT) can be a biomarker of higher affiliative and empathic behaviour in young females. Similarly, higher expression of testosterone (Testrn) in young males, in comparison to other age and sex groups, can increase vasopressin (VSP) and HPA activity that contribute to higher independence and, due to stronger adrenaline and cortisol oscillations, sensation seeking. Yet, both empathy and sensation seeking relate more to behavioural orientation and choice of reinforcers rather than to emotional valence. Arrows facing down indicate suppression, and arrows facing up – activation, of the release of the given hormone or neurotransmitter. GH, growth hormone; SOM, somatostatin; DA, dopamine; M/K(OR), mu/kappa opioid receptor systems; NE, brain noradrenalin; Estr, estrogen; Testrn, testosterone; OXT, oxytocin; VSP, vasopressin; HPA, hypothalamic–pituitary–adrenal axis. Horizontal arrows: activation of release, arrows down – suppression of release. The upper rectangle reflects Figure 1 composition.

There is good consensus in neurochemistry on the key role of endorphins binding to mu-opioid receptors (MORs) in positive emotional valence, physical and emotional pain relief (Leknes, 2008; Bodnar, 2018), relaxation, feeling of security, and affiliation (Barr et al., 2008; Curley, 2011). The action of MOR, activated by endorphins, suppresses the activation of the Hypothalamic-pituitary-adrenal (HPA) axis (i.e., suppresses mobilisation for an action) and induces the release of dopamine (DA) (Werling et al., 1988; Carr and Gregg, 1995; Degli Uberti et al., 1995; Yamauchi et al., 1997; Bodnar, 2018). There are several sites in the body and the brain that manufacture endorphins, and we can classify these sites into three groups (Figure 2).

**FIGURE 3:**
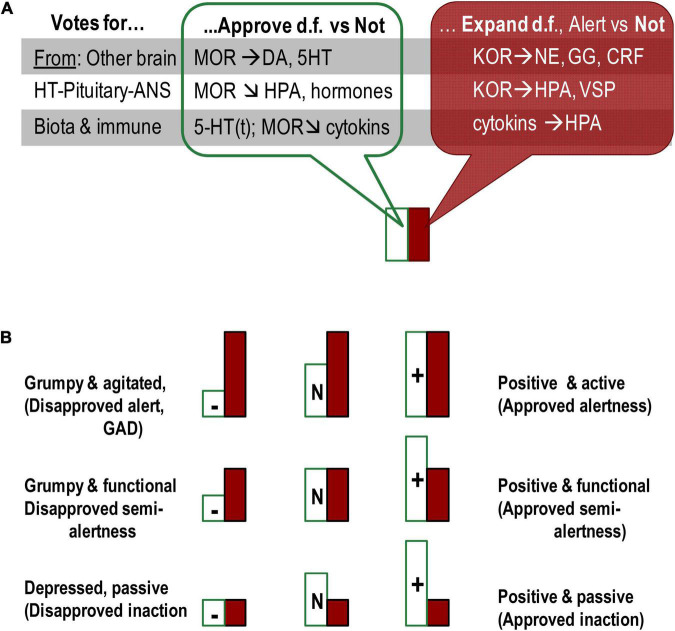
Presentation of emotional dispositions as summaries of the status of OR systems in three locations (gut microbiota, HT–pituitary complex, and ARAS-cortical complex). **(A)** All three locations contribute their “votes” for emotional dispositions, each using different neurochemical systems. **(B)** Combination of two types of emotional dispositions generates a diversity of emotionality-related consistent behavioural patterns. MOR-based dispositional approval (Satisfaction)-Disapproval induces (positive/negative) emotional valence whereas KOR- and cytokines-based Alertness-Indifference dispositions relate to expansion of behavioural degrees of freedom. Even though Approval and Expand systems (MOR and KOR) often suppress each other, they sometimes act in unison or independently of each other on other neurotransmitters. Moreover, KOR and associated activation systems (hormones and neurotransmitters) do not induce emotional valence. The Approval and Expand systems, therefore, are functionally distinct and do not represent one axis of either emotional valence or activation–deactivation. HT, hypothalamus; M/K(OR), mu/kappa opioid receptor systems; DA, dopamine; 5-HT(t), serotonin and tryptophan; NE, noradrenalin; GG, glutamate/GABA; CRF, corticotropin-releasing factor; HPA, hypothalamic–pituitary–adrenaline axis; VSP, vasopressin. Horizontal arrows: activation of release, arrows down – suppression of release.

1. Endorphins are produced by the bone marrow, in entanglement with the production of immune cells, and by gut microbiota, also managing the immune system (Stefano and Kream, 2008; Galland, 2014; Stefano et al., 2018). Experimental studies in psychoimmunology showed that bacteria *Mycobacterium butyricum* induces tonic secretion of opioid peptides (especially beta-endorphins binding to MOR) and decreases inflammatory pain (Bishai et al., 2009) whereas bacteria *Lactobacillus* induces the expression of MOR in the intestinal epithelial cells and mimic the effects of morphine (Cryan and Dinan, 2012; Dinan and Cryan, 2012; Lach et al., 2017; Ren and Lotfipour, 2020). This action was specific to neutrophils and not monocytes (immune cells) (Plein and Rittner, 2017), so the immune system alone could not be associated with the induction of positive or negative affect. When the amount of produced endorphins meets the demands for it from existing MOR density, an individual experiences the “gut feeling” of Satisfaction.
2. The cells producing endorphins in the anterior pituitary are located immediately next to the neurons producing stress hormones, which endorphins suppress. MOR suppress not only stress hormones but also gonadal (estrogens and testosterone) and social [oxytocin (OXT) and vasopressin (VSP)] hormones (Figure 3). Hormones regulate the state of the body related to fast-changing events, and peer interaction is full of such events. The suppressing action of MOR on hormones induces the sense of acceptance of chosen d.f. in behaviour, experienced as relaxation. MOR are also highly present in the vagus and other nuclei down the stream in the autonomic nervous system (Hamel and Beaudet, 1984; Nieuwenhuys, 1985; Peckys and Landwehrmeyer, 1999). This empowers MOR ability to control HPA axis arousal.

The other locations of MOR in the brain, which the endorphins bind to, were linked to behavioural homeostatic maintenance (Golgi cells in the cerebellum and in reticular formation) as well as motivational and programming processes [nucleus accumbens (NAc), ventral tegmental area (VTA), prefrontal cortex (PFC), amygdala (AM), HT, PAG] (Hamel and Beaudet, 1984; Nieuwenhuys, 1985; Peckys and Landwehrmeyer, 1999; Carlezon et al., 2009; Le Merrer et al., 2009). MOR, and also delta-opioid receptors (DORs), are G protein-coupled receptors (GPCRs) regulating the release of MAs, including the facilitation of dopamine release (Werling et al., 1988; Carr and Gregg, 1995; Degli Uberti et al., 1995; Yamauchi et al., 1997; Bodnar, 2018). Considering the “approval” effect of endorphins and their links to positive emotional valence, there is no surprise that these brain structures were noted for their involvement in motivation, planning, and anticipation. Limbic and cortical processing of the context of situations supplies the information about environmental needs and alternatives, and this information is always compared to the MOR-based summary on the homoeostasis of the body.

Emotional valence is seen here as the multi-“voting” summary from several endorphin locations managing the homeostatic balance of the body state. Positive emotional valence is experienced when the amount of endorphins corresponds to demands of MOR density, and negative (dysphoria, irritability) when this amount is insufficient, whether due to underproduction of endorphins or upregulation (increased density) of MOR receptors.

### Kappa-Opioid Receptor-Based Dispositions for Perceptual Alertness

Kappa-opioid receptors (KORs), despite belonging to the same neurochemical family as MOR, have significantly less effect on the valence of emotions in comparison to MORs. Instead of emotional valence, it is more accurately to associate KOR systems with sensory mobilisation processes activated to expand behavioural d.f. and to search for alternatives. Thus, KOR activation was linked to aversion, chronic anxiety, hallucinations and malaise, and activation of noradrenalin (NE) during chronic stress, but not to acute, situational stress, or specific phobias (Reyes et al., 2008; Schwarzer, 2009; Wittmann et al., 2009; Tejeda et al., 2012). There is significantly less KOR than MOR in the brain in general; however, the thalamus (anterior and lateral nuclei, i.e., the brain area integrating sensory information), has a significant presence of KOR in comparison to other brain structures (Hamel and Beaudet, 1984; Delay-Goyet et al., 1987; Mansour et al., 1987; Sharif and Hughes, 1989). Dynorphin that binds to KOR was found in GABA neurons in the central nucleus of the AM and lateral and medial hypothalamus (Tejeda et al., 2012), and high KOR density was also found in the lateral AM, HT, ventral striatum, and PFC (especially layer VI) (Mansour et al., 1987) (i.e., in the structures that, in addition to the thalamus, strongly contribute to perceptual and orientational mobilisation) (Hamel and Beaudet, 1984; Delay-Goyet et al., 1987; Mansour et al., 1987; Sharif and Hughes, 1989). KORs modulates NE release from the locus coeruleus (LC), contributing to the alertness in attentional and novelty-processing function of the NE system (Schwarzer, 2009; Bodnar, 2018), and, therefore, to perceptual sensitivity. This explains why in chronic stress (i.e., when KOR activation is above the optimal limit), people report an inability to focus and discomfort in novel situations, as an excess of NE release was also linked to “above optimal arousal” and inability to focus.

It has been shown that the same NE axons that respond to dynorphin have a co-localisation with excitatory glutamate (Glu) as well as stress-related corticotropin-releasing factor (CRF) (Reyes et al., 2007, 2008; Wee and Koob, 2010). Dynorphins that bind to KOR (and not other ORs’ peptides) appeared to be able to stimulate HPA axis activity, to increase adrenocorticotropic hormone (ACTH) *via* action on CRF (Bruchas et al., 2010) and cortisol release (Williams et al., 2003; Pascoe et al., 2008; Miller et al., 2018). This explains the involvement of KOR in alertness and (in clinical cases) dispositional anxiety.

The three groups of locations, including dispositional emotional valence, described earlier, also have neurochemical systems inducing dispositional alertness:

1. In the gut microbiota, the bacteria *Campylobacter jejuni* has been found to enhance anxiety-like behaviours in mice and extend its action all the way to the brain, activating the amygdala (Morais et al., 2021). Known pathogens such as *E. coli* (Dinan and Cryan, 2012; Farzi et al., 2018) or *Salmonella typhimurium* (Dunn et al., 2003) and others (Farzi et al., 2018) were shown to activate the HPA and brain NE release. There is a great variability in how bacteria affect the HPA system, from acting directly on the CRH secretion (Vakharia and Hinson, 2005), mimicing the proteins that are used by ACTH in pituitary (Farzi et al., 2018; Misiak et al., 2020), acting on the brain structures regulating the HPA activation (Haddad et al., 2002; Morais et al., 2021) or acting on inflammatory cytokines that could easily reach HT and other brain structures (Haddad et al., 2002; de Weerth, 2017; Salvador et al., 2021). The immune system alone, therefore, can activate the HPA axis inducing behavioural alertness, whereas gut microbiota has to couple with the circulating cytokines in order to affect HT and provoke a potent release of CRH (Haddad et al., 2002; de Weerth, 2017; Salvador et al., 2021).
2. KOR are present on the terminals of pituitary and hypothalamic neurons and affect the release of stress hormones and “social” hormones in this complex. ORs located on terminals of OXT and VSP neurones in the neurohypophysis are predominantly KOR, influencing the release of neurohypophysial NE (Zhao et al., 1988), CRF, and HPA axis activation (Bernstein et al., 1996; Bodnar, 2018). Since Empathy (EMP) is considered in the FET as a trait behavioural orientation trait based on OXT–VSP activity, KOR involvement in the regulation of these hormones can be seen as amplification of orientational aspects of behaviour.
3. As mentioned above, in the rest of the brain, KOR are present mostly in the structures related to behavioural orientation and information processing – thalamus, central AM, ventral striatum, and PFC.

Despite the association of KOR with NE and HPA arousal (Hauser et al., 2005; Tejeda et al., 2012; Bodnar, 2018), KOR action is very specific to dispositional chronic anxiety and stress but not to acute stress or specific phobias (Schwarzer, 2009). Thus, studies on animal models reported that KOR antagonists decrease avoidance and anxious dispositions (Schwarzer, 2009; Van’t Veer and Carlezon, 2013; Bodnar, 2018); however, KOR agonists don’t produce a transient elevation of anxiety. Instead, they produce perceptual distortions of sensory stimuli, depersonalisation, speech processing problems, and thought disorganisation (i.e., features of perceptual over-arousal) (Hauser et al., 2005; Tejeda et al., 2012; Van’t Veer and Carlezon, 2013). Moreover, only high doses of KOR agonists induce anxiety, while low doses act as an analgesic, and very low doses may induce positive mood states (Schwarzer, 2009; Bodnar, 2018). It would be, therefore, wrong to consider KOR as a system of negative emotional valence even though they are often in rival relationships with MOR and activate the HPA axis.

### Dispositional Emotionality Comes From the Supply/Demand Ratio of Endogenous Opioid Receptor Peptides

The production of opioid peptides and even the existence of their receptors appeared to be a very plastic process, opening a window for individual variability. The density of ORs can be increased (*upregulation*) or decreased (*downregulation* and desensitisation) depending on the supply of endorphins binding to them and the sensitivity of the receptors themselves. A single administration of opiates often triggers a set of changes that usually is restored by a chain of recovery mechanisms (Waldhoer et al., 2004; Christie, 2008; Carlezon et al., 2009; Schwarzer, 2009; Nagi and Piñeyro, 2011). Downregulation of receptors is observed mostly after chronic overuse/overproduction of these receptors’ agonists as a protective feedback mechanism, and upregulation develops in cases of consistent deficit of needed peptides (due to their under-production) (Waldhoer et al., 2004; Christie, 2008; Carlezon et al., 2009; Schwarzer, 2009; Nagi and Piñeyro, 2011). Moreover, there are factors other than just chronic overuse of opiates affecting up–down regulation of ORs, such as genetic dispositions or exposure to toxins, etc. (Waldhoer et al., 2004; Christie, 2008; Carlezon et al., 2009; Schwarzer, 2009; Nagi and Piñeyro, 2011). Individual differences in the production of opioid peptides and/or in the density of their receptors could, therefore, produce consistent individual differences in dispositional emotionality. These consistent individual differences are colloquially named in the FET as emotionality-related traits of the temperament of dispositional Satisfaction (based on MOR), Impulsivity (not discussed here), and Neuroticism (based on KOR).

The difference between *dispositional* emotionality and *situational* emotional experiences is that emotional dispositions are experienced before or regardless of the triggering events, whereas commonly known emotions are described as a type of reaction to specific situations (Trofimova, 2018). In contrast to that, hormonal and MA–ACh systems are involved more in the regulation of situational selection of d.f.: hormones as fast-acting regulators, more in control of the somatic systems that have to adjust to explicit and immediate features of events, and MA–ACh systems as regulators of behaviour related to not immediate, distant, and/or abstract features of events (probabilistic).

In line with the idea of a neurochemical continuum between temperament traits and symptoms of psychopathology (Sulis, 2018; Trofimova and Sulis, 2011; Trofimova, 2021a,b), weak biases in the ratio between the supply and demand of endogenous peptides binding to OR can be expressed as emotionality-related traits of temperament whereas strong biases – as psychiatric disorders. Figure 2 illustrates several scenarios of emotional dispositions based on MOR and KOR systems of emotional valence and alertness. Indeed, upregulation of MOR was linked to extreme dysphoria and irritability, as seen in borderline personality disorder and attachment disorders (Cacioppo et al., 2000; Christie, 2008; Leknes, 2008; Bandelow et al., 2010; Curley, 2011). In contrast, MOR downregulation can be experienced as dispositional, often negligent satisfaction with “life as is,” low productivity, high agreeableness, and dispositional positive mood. As noted above, dysregulation in KOR systems can lead to chronic alertness (anxiety, neuroticism) and, on the opposite pole – to dispositional indifference. Indeed, clinical levels of KOR dysregulation were linked to agitation, chronic anxiety, chronic stress, inability to relax, and behavioural arousal seen in generalised anxiety disorder (Hauser et al., 2005; Reyes et al., 2008; Schwarzer, 2009; Wittmann et al., 2009; Schindler et al., 2010; Wee and Koob, 2010; Tejeda et al., 2012).

## Decoupling Hormonal Systems From Emotionality

### Segregations Within Pituitary–Hypothalamic Systems Relate to the Regulation of Non-emotional Aspects of Behaviour

Traditionally, hormones are viewed as neurochemical systems of emotionality. Studies of the structure and functionality of the pituitary–hypothalamic complex, however, showed that this complex has well-defined morphological and functional segregation linked to body homoeostasis, largely unrelated to emotional regulation. The pituitary’s segregation, and hypothalamic projections to parts of the pituitary, support the idea of the differential regulation of physical vs. social aspects of behaviour, known as the activity-specific approach in temperament, which is one of the principles of the FET structure (Trofimova, 2016; Trofimova and Robbins, 2016; Trofimova and Sulis, 2016b; Rusalov, 2018). Indeed, there are groups of cells in the anterior pituitary generating hormones and neuropeptides which regulate the body’s physical endurance and metabolism (such as thyroid, growth hormone, and somatostatin), whereas so-called “social hormones,” OXT and arginine–VSP are manufactured by the magnocellular neurons hypothalamic supraoptic (SON) and paraventricular (PVN) nuclei projecting to the posterior pituitary (Vazquez-Borrego et al., 2018; Swaab et al., 2021).

Gonadal hormones regulated by the pituitary–hypothalamic complex (called the hypothalamic–pituitary–gonadal axis) have a distinct set of secretion sites and also represent an example of a system regulating non-emotional (sexual and reproductive) aspects of behaviour. Gonadal hormones, especially estrogen, regulate not only glands associated with sexual and reproductive functions but also the brain’s neurotransmitters and associated memory functions (noticeable in post-menopausal women) (Chavez et al., 2010; Lu et al., 2019) and behavioural plasticity (Almey et al., 2015; Lu et al., 2019) (i.e., also non-emotionality aspects of behaviour).

Finally, the HT PVN releasing corticotropin-releasing hormone/factor (CRF) as the component of the HT–pituitary complex was, for a long time, linked to emotional regulation due to its well-documented involvement in stress response. However, its involvement contributes more to the preparation of the body for sudden dramatic changes in behaviour and not even necessarily negative emotional valence of events. For example, HPA axis activation occurs during a sexual act between loving partners in very safe and trusting relationships, largely due to dehydroepiandrosterone (DHEA) produced by adrenal glands (Pluchino et al., 2015). Adrenaline/epinephrine is a substance acting on sympathetic ANS, and its effects are very diffuse, producing a generalised state of arousal. Contrary to the common view of adrenalin as primarily a stress hormone, studies from the 1960s show that the action of adrenalin is not specific to the fear reaction: adrenalin increases the emotion of anger in the presence of an anger-provoking stimulation, and a euphoric emotion in the presence of a euphoria-provoking stimulation (Schachter and Singer, 1962; Dror, 2016). As noted above, participants in these experiments reported “cold,” and not genuine emotions largely influenced by the context (“knowing” that they are probably afraid rather than being afraid in fearful situations and “knowing” that they are probably angry in anger-provoking situations). Adrenaline has since been recognised as a hormone preparing the body for an intense and immediate behaviour and providing behavioural arousal rather than inducing specific emotions (Schachter and Singer, 1962).

In this sense, here we point out that all parts of the HT–pituitary complex are more involved in the homeostatic regulation of the body’s state in fast-changing contexts rather than in mainly emotional regulation.

## Putting Three Neurochemical Families Together and Taking Them Apart

### Location Doesn’t Tell the Story: Lessons From Opioid Receptor and Biota-Based Systems of Dispositional Emotional Valence and Alertness

If we want to progress with the mapping of biomarkers of emotionality, traditional assignment of behavioural regulatory systems to brain structures and neuro-somatic locations should be likely reconsidered. It is important to note that brain structures differ in their functionality only because they consist of different chemical compositions. Functional roles of neurochemical systems, therefore, provide perspectives for analysing the involvement of brain structures in behavioural regulation. The action of opioid peptides is a clear example that it is not the location but the neurochemical system (presented in multiple locations) that has a distinct functionality in behavioural regulation. The examples are diffuse action of endorphins binding to MOR inducing dispositional emotional valence and action of dynorphins binding to KOR in dispositional alertness. All locations [gut microbiota, HT–pituitary complex, and ascending reticular activating (ARAS)-cortical complex] manufacturing and/or binding opioid peptides contribute their “votes” to emotional dispositions, each using different mechanisms and neurochemical partners (Figure 2).

The three groups of location for endorphin production all induce a sense of satisfaction, “approval” of current behavioural choices but with slightly different functionality. Guts endorphins give the dispositional sense of body’s comfort (or discomfort needing to be addressed) unrelated to the current events; HT–pituitary endorphins suppress hormones-based reactivity to fast-changing events, inducing the dispositional approval of current behavioural choices; finally, the brain MOR activity contributes to approval d.f. in actions or perceptions related to future, distant, or abstract events, i.e., more implicit elements of behavioural regulation. The last type of effect is seen in the improved mood while planning or dreaming of possible positive outcomes. The subjective feeling of satisfaction, approval, and acceptance can be seen in all activities linked to endorphins release – exercising, having meals, setting up plans, and following plans.

In contrast, KOR locations and entanglement with HPA arousal and NE release indicate KOR different functionality unrelated to emotional valence. The FET considers KOR systems associated with emotional dispositions for perceptual and behavioural alertness associated with expansion/search for alternative d.f. in behaviour. Such alertness works as an emotional, dispositional amplifier of orientation aspects of behaviour. Even in addiction studies, KOR systems have been implicated in ethanol-seeking behaviour, often comorbid with chronic anxiety (Wee and Koob, 2010; Tejeda et al., 2012), supporting the idea of their key role in perceptual arousal, amplifying behavioural orientation.

There are, therefore, functional differences in the effects of these two OR systems, which we can colloquially call MOR-based “satisfaction–dissatisfaction” (or “approval–disapproval”) and KOR-based “Alertness (expansion of d.f.)-indifference,” noticed in Neuroticism. MOR and KOR systems often act in opposite directions and suppress each other; however, they sometimes act in unison or independently of each other on other neurotransmitters (Pan, 1998; Bodnar, 2018). As noted, KOR do not induce emotional valence, and MOR system can add “sweeteners or bitterness” to both behavioural alertness and inactivity (as in relaxation) (Figure 2). These systems are, therefore, are functionally distinct and do not represent two polarities of one axis.

### Three Interacting but Distinct Classes of Neurochemical Systems Contribute Differently to the Selection of Degrees of Freedom in Action Construction

**FIGURE 4:**
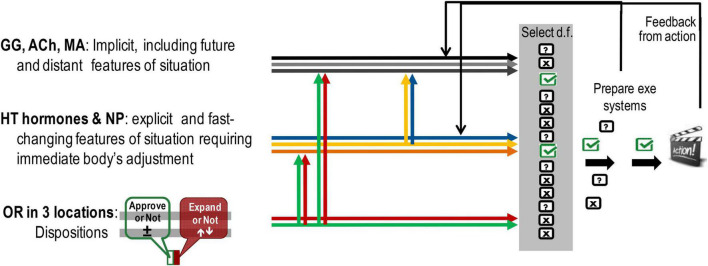
Differences in the influences on construction of action coming from the systems of dispositional emotionality, hypothalamic (HT)–pituitary systems of body adjustment, and hippocampal–cortical systems of information processing. Location-wise there is an overlap between OR systems and two other groups, however, as illustrated here, there are differences in their neurochemical composition and resulting functionality. GG, GABA/glutamate; ACh, acetylcholine; MA, monoamine neurotransmitters of the brain; NP, neuropeptides excluding endorphins and dynorphins; OR, opioid receptor and peptides in three locations: microbiota and immune system, HT–pituitary complex, and cortical-basal ganglia systems in the brain.

The roles of brain neurotransmitter systems – MA, ACh, Glu–GABA (GG) [not discussed here but reviewed earlier (Trofimova and Robbins, 2016; Trofimova, 2021a,b) differ from those played by OR and hormonal systems, as they provide a probabilistic assessment of events and generation of potential programmes of actions. In this context, it might be beneficial to separate at least three aspects of behavioural regulation that currently are considered as part of emotionality, but only one aspect can be viewed as true emotionality (Figure 4):

- OR-based dispositional emotionality as (in our view) true emotionality (Figure 4, lower group of arrows). MOR-based emotional valence gives a pro-approval or pro-disapproval bias to behavioural choices, with a range of states between satisfaction (positive affect) and dysphoria (negative affect) (green arrows in Figures 1, 3, 4). The KOR-based system provides alertness or indifference disposition that impacts the decision on whether or not the behaviour should change, and if so – increases perceptual sensitivity (red arrows in Figures 1, 3, 4). As noted by the OR systems, we refer to both OR and microbiota and understand that they represent very different systems.
- Hormone-based changes in the body as construction of action in response to fast changes and explicit aspects of events (Figure 4, middle group of arrows).
- Brain neurotransmitter-based cognitive appraisal and construction of action in response to a wide range of context, especially more implicit and not immediately present (Trofimova and Robbins, 2016; Trofimova, 2021a,b; Figure 4, upper group of arrows).

All three classes of neurochemical systems contribute to the selection of d.f. in actions. This selection is an interactive process, influenced by biases from OR-based emotional dispositions, the hormone-based situational status of the body and context-processing managed by brain neurotransmitters. The feedback at the preparation and execution stages of action affects the hormonal and brain neurotransmitter’s regulation of behaviour (middle and upper groups) but has a less immediate impact on emotional dispositions.

Also, as reflected in the title of the FET framework, all three classes of neurochemical biomarkers work in ensembles.

First of all, OR receptors represent the class of GPCRs regulating the release of all main neurotransmitters and many hormones. Therefore, they affect non-emotionality aspects of behavioural regulation regulated by brain neurotransmitters. As summarised in other FET-related reviews (Trofimova, 2021a,b), MORs induce DA release in VTA–NAc and suppress NE release, whereas KOR action can lead to the opposite pattern (Degli Uberti et al., 1995; Carlezon et al., 2009; Wittmann et al., 2009; Tejeda et al., 2012; Bodnar, 2018). Functionally, this means that the KOR system can pause the DA-based integration of actions but boost the NE-based search for novel alternatives, whereas MORs can suppress this new search (also the contextual analysis) and “approve” the existing alternatives in actions. There is also a strong neurochemical cooperation between the MOR system and the serotonin system, which is also prominent in the homeostatic maintenance of behaviour. It has been shown that MOR (but not other ORs) agonists inhibit 5-hydroxytryptamine (serotonin) (5-HT) in the hippocampus (HC) (Yoshioka et al., 1993). In our experiments, when pregnant rats were chronically exposed to opium that caused downregulation of MOR receptors, they also had a significant decrease in endurance in four types of maternal behaviour, a decrease of 5-HT in HC, a decrease of BDNF and an increase of corticosterone (stress hormone) (Rezaei et al., 2021). Also, out of all OR, only MOR develop heteromeric complexes with the 5-HT1 receptors (Fujita et al., 2014).

If OR and microbiota induce dispositions unrelated to the features of situations, hormonal systems are more tuned to the features of situations, regulating the body’s state related to fast-changing and explicit features. Although the OR and hormonal systems interact, they can be still seen as functionally distinct. As the main trend, OR systems suppress hormonal release, whether it is gonadal (Liu et al., 2011; Ho, 2019), social (Smith and Wise, 2001; Loth and Donaldson, 2021), or stress hormones. Gonadal hormones, in turn, have a way to modify OR binding and density, also in a very specific pattern. Estrogen can internalise MOR in cell groups of the limbic-hypothalamic complex, and this modulates sexual receptivity (Micevych et al., 2003; Huhn et al., 2018). There are, however, exceptions to this trend, indicative of more specific functionality of OR systems than just universal “suppressors.” Thus, KOR can activate VSP release (Zhao et al., 1988; Bernstein et al., 1996; Bodnar, 2018). Also, MOR density is higher in females than in males, indicative of MOR-estrogen interactions (Huhn et al., 2018). Moreover, while estrogen decreases KOR-binding dynorphins expression in the arcuate nucleus of the HT, testosterone increases dynorphins in the supraoptic nucleus of HT and bed nucleus of stria terminals (Becker and Chartoff, 2019).

These interactions between three classes of neurochemical systems implicated in emotionality can explain the observations of emotional dispositions being capable of affecting all non-emotional aspects of behavioural regulation – orientation, integration of a programme of actions, energetic maintenance (green and red arrows in Figures 1, 3, 4). As seen above from the general impact of MOR action and also from its cooperation with 5-HT, MOR-based Satisfaction/Approval systems amplify the sense of homeostatic maintenance balance; KOR-based systems of Alertness-Expansion (known as Neuroticism) amplify orientation aspects of behaviour. Impulsivity (not discussed here) can be seen as an amplification of the integration and initiation of actions.

Finally, cognitive appraisal (emerging from the activity of brain neurotransmitter systems involved in contextual processing) is often seen as part of emotionality (Schachter and Singer, 1962; Dror, 2016). Indeed, social emotions are experienced differently in those contexts where an individual was either an agent or a victim of harm (Kanen et al., 2021), illustrating the impact of the upper group of neurochemical systems in Figure 4. Moreover, evaluative biases stemming from emotional dispositions have been shown to affect all kinds of perceptual processes. This led to the theory of somatic markers (Damasio, 2000) and embodiment in cognition, which we first named as the “phenomenon of projection through capacities” when we found this effect in the late 1990s (Trofimova, 1997, 1999). These evaluative biases impact even the highest levels of cognition, such as attribution of meaning to emotionally neutral, abstract words (Trofimova, 1999, 2014). In our experiments, we demonstrated the existence of cognitive biases associated with temperament traits and sex (i.e., neuro-chemically based individual differences) of healthy participants affecting their highest form of cognition (meaning attribution). Temperament traits of high physical and social endurance, faster tempo in motor and verbal tasks were found to be associated with a positivity bias, whereas the traits of neuroticism, high emotionality were associated with a negativity bias in estimations of neutral abstract and very common concepts (Rusting and Larsen, 1998; Trofimova, 1999, 2014; Zelenski and Larsen, 1999). Interestingly, participants with high intellectual endurance (sustained attention) and with high probabilistic thinking had a significant negativity bias in their estimations of abstract common words. This is consistent with reports that MORs suppress brain ACh release, which, in turn, is the major neurotransmitter regulating sustained attention and probabilistic processing (Trofimova, 2019). Moreover, in line with the ETS theory, males in three different cultures had a positivity bias toward sensational concepts and evaluative criteria, whereas females favoured ordered and predictable objects (Trofimova, 2012, 2013, 2014).

These effects point to the impact of hormonal systems regulating social perception and underlying sex differences on perception in general, which can or cannot be associated with emotional valence.

### Previous Views on the Role of Dopamine Systems in Positive Emotionality Missed the Mediation by Mu-Opioid Receptor and Delta-Opioid Receptor Systems on Dopamine Release

The analysis of neuroimaging studies investigating neuroanatomic biomarkers of positive and negative affects showed no significant correlations between brain areas and positive affectivity (Kaufmann et al., 2019). Moreover, studies of psychiatric disorders associated with negative affect showed that practically all brain structures were involved in negative emotions. This lack of specificity of brain structures in emotional valence highlights the need to focus more on their neurochemical rather than neuroanatomic biomarkers.

Neurochemical hypotheses, however, can also have flaws, and one of the examples of common misconceptions is the attribution of positive emotional valence to the direct action of DA. Meanwhile, the release of DA in ventral striatum (so-called “rewards circles”) is controlled and facilitated by MOR activation as part of a GPCR mechanism dominating MA release. The “reward circuits” include the VTA and the NAc, which indeed are rich in DA and are highly involved in addiction. To disentangle the functional roles of MOR and DA in positive emotionality we can look at the effects on DA release when MOR systems are not involved.

First of all, the release of DA was more consistently associated with a reaction to the significance, saliency, of stimuli rather than to positive emotions. Thus, activation of DA neurons in the VTA, was found to be similar whether it was induced by negative experiences (prolonged stress) (prolonged stress) (Ortiz et al., 1996; Reynolds and Berridge, 2001; Carlezon and Thomas, 2009), the anticipation of aversive stimuli (Ortiz et al., 1996), or, as well-known, pleasurable experiences (psychoactive drugs). Some studies report that treatments that increase NAc excitability depress mood, whereas treatments that reduce this excitability appear to elevate mood (Carlezon and Thomas, 2009). Second, the DA hypothesis of positive emotional valence was confronted with non-supportive reports (Jensen et al., 2003; Saal et al., 2003). Appetitive stimuli appear to enhance activity in the mesocortical DA system to *a lesser and not higher degree* and for a more transient period than did aversive stimuli (Robbins and Everitt, 1996; Jensen et al., 2003; Saal et al., 2003; Seamans and Yang, 2004). Higher DA release was reported not only in positive but also in negative circumstances, such as a defeat, aversive stimuli, stress, and foot shock (Robbins and Everitt, 1996). Third, “reward circuits” were reported to be activated during anticipation of getting a reward but not at the receipt of the reward (O’Doherty et al., 2002), suggesting that they process the projection of future events rather than being responsible for inducing specific (positive) emotional valence. Finally, addiction itself appeared to be not driven by the positive affect but by the damaged habit system (drug addicts often report that they don’t like their drugs but just can’t stop themselves) (Everitt and Robbins, 2016).

Earlier, we pointed out the more consistent roles of DA in the regulation of the assignment of priorities, salience and sequencing to behavioural elements necessary for behavioural plasticity and integration of actions (Trofimova and Robbins, 2016; Trofimova, 2021a,b). This is in consensus with the position of other authors (Seamans and Yang, 2004; Yin and Knowlton, 2006; Tepper et al., 2007; Seamans and Robbins, 2010; Graybiel and Grafton, 2015; Haber, 2016) and specialists in DA research pointing to the flaws of the “dopamine theory of extraversion and positive affect” (Robbins and Everitt, 1996; Seamans and Yang, 2004; Robbins, 2018). Dysregulation within the DA system was indeed found less associated with changes in emotionality and more so with a compromised ability to integrate behaviour adequately to a situation (i.e., impulsivity, rigidity, OCD) (Szechtman et al., 1998; Seamans and Yang, 2004; Robbins, 2010, 2018). The remarkable role of DA in “salience-labelling” can be seen in its association with a pathological attachment of importance to irrelevant stimuli in schizophrenia (Gray, 1998; Coyle, 2006) and psychoticism (Kapur, 2003), both linked to an excess of DA but both associated with negative, and not positive, affectivity. Meanwhile, some studies of DA D4 receptor genes found no associations with Extraversion (Munafo et al., 2008). Moreover, the early opinion that Extraversion (or general arousal) was based on the cortical-ARAS system was confronted by experiments in neurochemistry that showed that the ARAS system has at least four different sub-systems of arousal, diverging in their functionality, and directionality of neurotransmitter release (Robbins and Everitt, 1996; Trofimova and Robbins, 2016). In line with other researchers (Ruhé et al., 2007), the FET framework suggests that neither DA nor other MAs (NE, serotonin, 5-HT) directly regulate moods. Instead their release during emotional experience and commonly described MA roles in emotionality-based psychopathology (for example, Liu et al., 2018), are, therefore, secondary to the activation of OR systems that regulate MA release. Positive or negative emotional valence is so far more consistently associated with the effect of mu-ORs acting on MA as part of a GPCR mechanism (Trofimova and Robbins, 2016; Trofimova, 2021a,b), rather than the effect of release of monoamines *per se*.

Brain neurotransmitter (NT) systems work in ensembles with hormonal and OR systems on the selection and composition of a behavioural act (as depicted in Figure 4). OR systems provide the body’s bias for decision making on “what’s next” in behaviour (“accept vs. not-accept,” “change-search vs. not-change”); hormonal systems provide the tuning of the body to immediately present and fast-changing aspects of events, whereas brain neurotransmitters deal with not immediately present, abstract, and probabilistic aspects of events. What is called “emotionality” is, therefore, presented here as a multi-component process resulting from the “voting” of many body and brain systems on the prognosis of events. Future research, therefore, can focus on disentangling the roles of these three neurochemical families in behavioural regulation by controlling for biases stemming from the action of OR and hormones on brain NTs during emotionality-related experiments.

### Sensation Seeking Is a Temperament Trait That Is Related More to Behavioural Orientation Than Emotionality

#### Noradrenaline, Adrenaline, and Under-Arousal of Sympathetic ANS Are Involved in Sensation Seeking

Sensation Seeking (SS) is a temperament trait describing a drive for risky and extreme experiences associated with physical sensations; SS is most prominent in young males (Zuckerman, 1994, 2007). Sensation seekers are often but not always involved in experimenting with drugs and very risky activities such as speeding on highways. Initially, Zuckerman, who identified SS as a biologically based trait, first suggested (and later revised it) that MAO inhibitors, and especially a deficiency of the NE metabolite called MHPG, are the leading players in this trait (Daitzman et al., 1978; Zuckerman, 1994, 2007; Longato-Stadler et al., 2002). MAO acts on both DA and NE, and studies in animals indeed showed the biological basis of the SS trait, linking it to DA and corticoid systems (Dellu et al., 1996; Stansfield et al., 2004). DA and NE share the same regulatory protein systems, and, therefore, it is not surprising that NE was also implicated in SS.

Considering the key role of NE in perception and attention to novelty, it could be seen that low baseline noradrenaline and adrenaline can generate a subjective feeling of insufficient perceptual arousal. Lower than average MAO activity was indeed reported in professional bullfighters (Allison et al., 2012), professional risk-takers (Carrasco et al., 1999), and male criminal subjects (Longato-Stadler et al., 2002). Deficiency in the NE transporter was linked to drug-seeking and higher consumption as a way of self-stimulation (Weinshenker and Schroeder, 2007). As noted, addiction research showed that NE and KOR systems are involved in drug-seeking behaviour (Weinshenker and Schroeder, 2007; Wee and Koob, 2010; Tejeda et al., 2012), which is common in sensation seekers.

However, MAO inhibitors acting on brain NE systems might not be the only factors in SS. Thus, when analysing more specific SS scales, several researchers, including Zuckerman himself, noticed that it is mostly the scale of Disinhibition (measuring impulsivity) but not the Boredom Susceptibility or Thrill Seeking scales which had associations with MAO deficiency (Carrasco et al., 1999; Longato-Stadler et al., 2002; Zuckerman, 2007). Adrenalin (A), which is the end product of noradrenaline, was also implicated in SS (Zuckerman, 2007; Allison et al., 2012; Hinnant et al., 2016). NE can be transformed to adrenaline, or NE projections from LC can activate adrenaline in the ganglia of the sympathetic ANS as part of HPA axis arousal. Either way, NE and A have very tight relations, with NE more involved in cortical processes and with A more involved in the HPA axis.

The action of A and associated stress hormones prepare the body for drastic differences in behavioural alternatives: changing the heart rate, blood pressure, suppressing digestion and even the performance of routine actions. When the cortical NE system of orientation underperforms (as a result of dysregulation of MAO inhibitors or other factors), the hypothalamic–pituitary, A-based system takes over, inducing an inability to focus and high impulsivity that is well known in ADHD. The under-performance of the ANS and adrenaline system can induce a feeling of insufficient arousal, generating a need for dramatic, stimulating experiences, thrill-seeking that would activate the underperforming HPA axis. Indeed, in adolescent males, sensation seeking was associated with lower resting heart rate (Kavish et al., 2017) and lower sympathetic ANS reactivity (Allison et al., 2012; Hinnant et al., 2016). In fact, Zuckerman himself called sensation seekers “adrenaline junkies” (Zuckerman, 2007).

#### The Role of Testosterone and Cortisol in Sensation Seeking

Stress reactivity or under-reactivity/underarousal (such as in SS) is commonly discussed (including the section above) with “stress hormones,” such as cortisol and adrenaline. However, it appeared that there is an interplay between stress hormones and gonadal hormones in the regulation of sensation seeking and empathy traits.

Remarkably, the association of SS with NE and A, or low sympathetic ANS arousal, was found only in young males and not females (Mehta et al., 2015; Harden et al., 2018). Statistically speaking, SS has a strong behavioural sex dimorphism (males have several times more SS-related activities and accidents than females) (Wilson and Daly, 1985; Kruger and Nesse, 2004; Zuckerman, 2007), implying a role for testosterone in SS. Sensation seeking was indeed linked to elevated testosterone (Op de Macks et al., 2011; Peper et al., 2013; Mehta et al., 2015; Harden et al., 2018) and low cortisol (Netter, 2006, 2018; Mehta et al., 2015) much more in young males, in comparison to females. There is also a mutual regulation between NE/A and testosterone that can explain the higher rates of addiction and risk-taking in males in comparison to females. NE and adrenaline, but not DA or 5-HT, stimulate testosterone release in both animals (Mayerhofer et al., 1992) and humans (Chichinadze and Chichinadze, 2008; Hart et al., 2011). Vice versa, testosterone increase was associated with higher NE release (Lara et al., 1985; Jones et al., 1998). Both testosterone and estrogen reduce levels of MAO inhibiting monoamines, and this leads to increased levels of monoamines, in our case – NE (Hart et al., 2011). However, some authors noted that testosterone is much more involved in SS than estrogen (Maattanen et al., 2013; Peper et al., 2013; Harden et al., 2018). Since FET associates NE with the regulation of orientational aspects of behaviour, the SS can be also viewed as a temperament trait of behavioural orientation inducing bias toward sensational and risky activities.

There have also been consistent reports associating SS with low cortisol (Piazza et al., 1993; Wang et al., 1997; Rosenblitt et al., 2001; Netter, 2004, 2021; Tyrka et al., 2007). Similarly to testosterone, the association of low cortisol with SS was observed only in males (Rosenblitt et al., 2001). In combination with the findings of sympathetic ANS under-arousal in the SS, this points to a possible underperformance of the adrenal glands, where both cortisol and adrenaline are produced as regulated by the pituitary (Netter, 2006, 2018).

Low cortisol and adrenaline might be a consequence of the overactive pituitary, which also produces gonadal hormones and so could increase the level of testosterone. Considering the daily oscillations of cortisol, it is not the steady low or steady high amount of hormones in an individual’s nervous system that could generate occasional cravings for stimulation. Instead, it is likely the size of the amplitude of hormonal cycles that, in moderate amplitudes, generate healthy curiosity, orientation, and exploration behaviour. This resembles the ancient concept of chemical imbalances, applicable to modern neurochemistry: the optimal range of variations in neurochemical systems that mutually regulate composition and decomposition of each other’s components would generate a healthy SS temperament trait (highly exploratory behaviour oriented to boost behavioural arousal). When these chemical cycles go to more extreme amplitudes, however, this could lead to semi-clinical CPB, and in this case – a strong feeling of boredom and, as our patients say, “a need for something dramatic.”

The involvement of cortisol in humans (and corticosterone, or CORT in animals) provides anti-inflammatory and nutritional actions. Therefore, similar to serotonin, cortisol, and COR most likely play a role of homeostatic maintenance of the body when the amplitudes of natural cycles swing to dangerous extremes. Microbiota provide a source of elements (including tryptophan) regulating HPA arousal and suppressing cytokines (Haddad et al., 2002; Dunn et al., 2003; Vakharia and Hinson, 2005; de Weerth, 2017; Farzi et al., 2018; Misiak et al., 2020; Morais et al., 2021). There are, therefore, several factors protecting the nervous system from over-heating by constant orientation and stress, which include not only brain but also immune, gut biota, and endocrine body systems. These systems consist of cells and micro-organisms constructing their sustainability and survival by participation in bodily functions. External behaviour is only a small part of these processes. However, to understand individual differences in observable behaviour, we should respect the interactions between these internal biochemical systems, and not just the interactions between brain areas.

Due to the variability and complexity of these mechanisms, there are, therefore, not one but multiple scenarios when microbiota-HPA-HGA relations could be shifted to particular behavioural patterns, such as SS or neuroticism.

#### A Mix-up Between Sensation Seeking, Novelty Seeking, and Reward Sensitivity

Despite the reports confirming the association of high NE, high testosterone and low or unsteady cortisol and adrenaline with SS, there have been several studies reporting no such associations (Netter, 2021). One reason would be the use of different concepts and associated tests: SS, Novelty Seeking (NS), and Reward Seeking (RS). We had a chance, in separate and extensive interviews with Cloninger (1993) and Zuckerman (2007), to ask them about the differences between their SS and NS concepts. Both authors thought that the concepts were different, suggesting that SS is more driven by hormones and NE systems, as seen in addicts and thrill-seekers. In contrast, NS mainly referred to a need for novelty in perception, mainly regulated by NE rather than by cortisol, adrenaline, or testosterone (as in SS). NS doesn’t necessarily include a search for risky experience inducing an adrenaline rush but involves a cognitive component to a higher degree than in SS. The orientation to physical sensations is observed in addicts and people with risky hobbies (Zuckerman, 1994, 2007), including parachutists and bullfighters (Allison et al., 2012), in which novelty is often minimal. The difference between SS and NS concepts and also the differences between the tests measuring these traits, therefore, can explain why studies using Cloninger’s NS scale found positive associations between NS and baseline plasma NE but not with testosterone (Wang et al., 1997; Gerra et al., 1999).

The second reason for the inconsistency in the neurochemical studies of SS is the use of samples of mixed ages, such as in the large study of Gerra et al. (1999), that used participants aged 19–60. SS, however, is most prominent in young males and, as Zuckerman (2007), decreases after the age of late 20s. This means that not just sex but also age can be a factor in SS. When a study mixes young and older groups, therefore, it might wipe out the significance of effects that are prominent more in younger groups.

Another mix-up happened when researchers equated SS with RS, measuring RS as the degree to which an animal or human performs a self-administration of drugs or actions leading to pleasurable stimuli. RS behaviour was linked to so-called dopaminergic “reward circles” that manage the compositions of a programme of actions. Systems. There has also been a trend to assume that SS is also a DA-based trait and might be a part of extraversion (Depue and Collins, 1999). Meanwhile, as summarised in detail earlier (Trofimova, 2021a,b), DA systems regulate primarily prioritising and integrating aspects of behaviour (plasticity, tempo, impulsivity) rather than SS or RS. The integrative role of DA can explain why DA systems are activated in the self-administration of drugs or approach-like actions: all these actions should be integrated and so involve DA release. In contrast to RS, the SS trait relates to orienting aspects of behaviour, a choice of specific reinforcers (that would activate the HPA axis of the individual) that affect the subsequent choice of actions. An additional set of arguments against the DA theory of SS is that SS has a strong sex dimorphism (males have several times more SS-related accidents than females) (Wilson and Daly, 1985; Kruger and Nesse, 2004; Zuckerman, 2007) with a well-documented role of testosterone in SS. In contrast, the sex differences in DA exist mainly in the distribution but not the release of this NT. These differences are subtle and cannot explain the sexual dimorphism in SS. Besides, high SS is associated not only with the male sex but also with younger age, and the rates of decrease in SS coincide with the rate of decrease in testosterone but not in dopamine (Trofimova, 2015).

Moreover, several authors pointed out the consistent absence of significant associations between Cloninger’s RS, NS scales, and the DA system (Vandenbergh et al., 1997; Robbins, 2018). There were inconsistent reports of either positive or negative associations with DA, and many studies reported the absence of an association between RS and NS and the D4 dopamine receptor gene (Vandenbergh et al., 1997; Kuhn et al., 1999; Ekelund et al., 2001; Jonsson et al., 2002; Strobel et al., 2003; Kim et al., 2006; Munafo et al., 2008). A recent investigation of a polymorphism in the gene encoding cathechol-*O*-methyltransferase (COMP, that regulates DA and NE) on SS, found no differences in males but a significant role of one COMP polymorphism in female SS (Schmitt et al., 2007). Another experimental study showed that neither testosterone nor estrogen had a significant association with RS, but testosterone was positively, and estrogen – negatively associated with SS (Harden et al., 2018).

There is emerging evidence that gonadal hormones can act on DA in opposite directions and in opposite parts of the striatum [i.e., the brain structure that integrates a programme of actions (behavioural sequences)]. The rise of testosterone in adolescents was found to be associated with a decrease in DA activity, whereas estrogen was associated with an increase in DA activity (Sinclair et al., 2014). Also, the differential impact of gonadal hormones on DA was not universal across brain areas. Testosterone was associated with increased ventral striatum activation (Op de Macks et al., 2011) and decreased dorsal striatum activation based on DA release (Purves-Tyson et al., 2014), whereas estrogen increased DA release in the dorsal striatum (Becker, 2000; Sinclair et al., 2014).

Such preferential activation of ventral and inhibition of dorsal striatum by testosterone can be put into perspective by research showing the involvement of the ventral striatum with the early stages of programming of behaviour, during the novel and complex tasks, and the dorsal striatum with more automatic integration of actions, when the task was well-learned (Everitt and Robbins, 2013; Graybiel and Grafton, 2015). This suggests that at least in adolescents, testosterone suppresses the integration of routine behaviour regulated by the dorsal striatum (unrelated to emotionality) and boosts motivational aspects of behaviour that are processed in the ventral striatum. Estrogen, on the other hand, facilitates the integration of well-learned behaviour, including the assistance of acetylcholine-based maintenance of habits by dorsal striatal and cerebellar networks and memory processes (Chavez et al., 2010; Lu et al., 2019).

The differential pattern of gonadal-DA interaction in individuals with different levels of testosterone and estrogen is consistent with the findings of sex differences in semantic perception: young males reported having more positive estimations of sensational objects, whereas young females do so for more ordered and routinised objects (Trofimova, 2012, 2013, 2014). The sexual dimorphism in behavioural abilities, disabilities, semantic perception and temperament traits, including sensation-seeking, are in line with the Evolutionary Theory of Sex (ETS) proposed in the 1960s, which explained sex dimorphism by an evolutionary systemic tendency for species’ self-regulation (Trofimova, 2015).

In sum, SS is considered in the FET as an orientation-related trait rather than an emotionality-related trait. The SS relates to behavioural orientation, i.e., choice of behavioural alternatives promoting access to psychostimulants, risky, and sensational activities. Neurochemically, the SS was linked to a combination of high testosterone, low cortisol and fluctuations in adrenaline levels inducing the HPA axis under-arousal.

### Empathy Is a Temperamental Trait That Is Related More to Behavioural Orientation Than Emotionality

Another trait that relates to behavioural orientation to specific reinforcers and that is often considered as an emotionality trait is EMP. Neurochemically, empathy is linked to hormonal systems, namely “social hormones,” such as OXT and arginine–VSP, that are manufactured by the magnocellular neurons in the hypothalamus and project to the posterior pituitary (Vazquez-Borrego et al., 2018; Swaab et al., 2021). Unlike “stress hormones,” reliably linked to the arousal component of emotionality, “social hormones” by themselves showed significantly less association with an emotional valence or emotional arousal. OXT was shown to induce a calming effect on HPA axis arousal (Quintana et al., 2016), including in association with estrogen (Ochedalski et al., 2007; van Anders et al., 2011; Gabor et al., 2012; McCall and Singer, 2012), but not necessarily positive emotions.

More consistently, OXT has been reported to have an association with pro-affiliative perception (van Anders et al., 2011; McCall and Singer, 2012; Decety et al., 2014; Shamay-Tsoory and Abu-Akel, 2016; Kanat et al., 2017; Jones and Sloan, 2018; Declerck et al., 2020). The neurochemical framework FET (Trofimova, 2016, 2021b; Trofimova and Robbins, 2016) summarised the consensus in the literature on the key role of OXT–VSP (in specific combination with gonadal hormones) in *empathy* (i.e., social type of behavioural orientation to other’s people motives and psychological states). Other authors also support the “social salience hypothesis of oxytocin” (reviewed in Shamay-Tsoory and Abu-Akel, 2016; Liu et al., 2019), underlying the tendency for OXT administration to increase pro-social perception, including trust (Declerck et al., 2020), face recognition (Decety et al., 2014; Kanat et al., 2017), facilitation of mother-infant interactions (Jones and Sloan, 2018), processing of social value (Liu et al., 2019), empathy, and perceived attachment (Heinrichs and Domes, 2008; Bartz et al., 2011, 2015, 2019; Meyer-Lindenberg et al., 2011; Olff et al., 2013; Kanat et al., 2014). The specificity of OXT in social orientation was demonstrated in cooperative games, showing increased cooperation after OXT administration when social information was present but reduced cooperation when it was not (Declerck et al., 2010). In support of the view on empathy as a social perception trait, OXT receptors are most present in those brain structures related to sensory-informational processing and behavioural orientation, [i.e., in the central nucleus of the solitary tract (NTS), central amygdala (cAM), septal nuclei, HC, insula, and prefrontal (both, medial and orbital) and cingulate cortex (Huber et al., 2005; Karelina and Norman, 2009; De Dreu et al., 2010; Viviani et al., 2011; Knobloch et al., 2012; Boccia et al., 2013; Baribeau and Anagnostou, 2015; Mitre et al., 2016; Quintana et al., 2016; Sabihi et al., 2017)]. Behavioural orientation is the consideration of d.f. in behaviour and the choice of reinforcers that an individual would use as feedback during the construction of subsequent actions. The presence of OXT receptors in these brain structures make them responsive to the social/affiliative features of situations in addition to other information processing.

Brain NE, the monoamine neurotransmitter that is consistently linked to orientational aspects of behavioural regulation (especially attention to novelty), also has a way to regulate OXT. Indeed, OXT and VSP magnocellular neurons have direct projections from the NTS, the centre of sensory integration of the state of somatic systems, and 80% of NTS to SON projections are NE-gic, putting the OXT system right at the core of social perception (Karelina and Norman, 2009). These associations can explain why high-NEU people have a higher sensitivity to social approval/support, in comparison to low-NEU, indicative of a possible dysbalance between (insufficient) endogenous OXT production and (likely high) OXT receptor density. In terms of situational emotions, neuroticism as negative dispositional expectations of outcomes, including expectations of low social support, can induce higher readiness to admit guilt and develop shame. As noted, OXT functionality is consistently associated with orientational (socially relevant) aspects of behaviour.

Oxytocin and VSP often down-regulate each other to balance their effects (Legros, 2001) and show opposite action in the central AM: OXT calms HPA arousal by acting on GABA neurons in the cAM (Huber et al., 2005; Viviani et al., 2011; Knobloch et al., 2012; Quintana et al., 2016) and suppresses VSP-responsive neurons (Meyer-Lindenberg et al., 2011; Viviani et al., 2011; Liu et al., 2019; Grinevich and Neumann, 2021). VSP is also involved in social perception but as a support of self-image in a social context. It is likely that their differences in the regulation of the orientation to others vs. orientation to self were reinforced in evolution by coupling with gonadal hormones: OXT has mutually supportive relations with estrogen, whereas VSP has with testosterone. There are consistent reports that the locations of OXT and VSP receptors in brain areas do not overlap, suggestive of their different functionality (Stoop, 2012). Moreover, VSP increases alertness for external stimuli by activating the central AM, increases sympathetic output, and decreases parasympathetic output (Huber et al., 2005; Knobloch et al., 2012). This contrasts with the calming action of OXT on central AM, increasing parasympathetic and decreasing HPA arousal.

Oxytocin and VSP are not, however, always rivals, coordinating their common task of social perception. They use each other’s receptors (Song and Albers, 2018), and VSP was also linked to empathy (Tabak et al., 2015). This might explain why, despite being called the “love hormone,” intranasal OXT administration reportedly also produces “antisocial” effects, such as elicitation of ethnocentrism (Zhang et al., 2019), racial bias (Sheng et al., 2013), or increased dishonesty toward outgroup members (Radke and de Bruijn, 2012; Shalvi and De Dreu, 2014; De Dreu et al., 2016; Daughters et al., 2017).

There is a balancing interaction between social and gonadal hormones, so a pro- or anti-social orientation shouldn’t be attributed to the OXT–VSP pair alone. It has been shown that a testosterone–oxytocin imbalance can induce either empathy (and social sensitivity, when OXT release is more active than testosterone release) or autism (and self-centred perception, in the opposite case) (Montoya et al., 2012; Crespi et al., 2016; Procyshyn et al., 2020) and even aggression (van Anders et al., 2011). Variations in the OXT gene indeed were linked to antisociality in adolescent boys (Hovey et al., 2016); however, this sex-age group is also known for having high testosterone, low cortisol, and dopamine (Rosenblitt et al., 2001; Sinclair et al., 2014). Also, VSP is released more in male-typical behaviour, such as pair-bond formation and competition (seen in aggressive actions) (Heinrichs and Domes, 2008; de Vries et al., 2012), as well as protective aggression (Heinrichs and Domes, 2008; Kanat et al., 2014). Maternal aggression is also correlated positively with OXT release in PVB and cAM (de Vries et al., 2012).

The ability of central OXT to inhibit HPA axis activity appeared to be dependent on circulating estrogen (Ochedalski et al., 2007; van Anders et al., 2011; Gabor et al., 2012; McCall and Singer, 2012) whereas central VSP appeared to be more dependent on testosterone (van Anders et al., 2011; Gabor et al., 2012; McCall and Singer, 2012). In fact, the role of VSP in self-centred and competitive behaviour was found in males and not females (de Vries et al., 2012; Uzefovsky et al., 2012). The imbalanced ratio of high testosterone combined with low cortisol was also found to be a factor in socially aggressive behaviour (Popma et al., 2007; Terburg et al., 2009; Montoya et al., 2012; Ponzi et al., 2016; Knight et al., 2017; Grotzinger et al., 2018). Also, as highlighted by Crespi (2016), testosterone exhibits opposite effects from OXT on diverse aspects of cognition and behaviour, most generally by favouring self-oriented, egocentric and non-social attention and information processing. Too much OXT can be associated with mental disorders such as social psychosis and schizophrenia if this (high OXT) is combined with low testosterone (Dinsdale et al., 2013; Crespi, 2016; Dinsdale and Crespi, 2017); an opposite pattern (high testosterone and low OXT) is also associated with psychopathology (autism) (Crespi, 2016; Crespi et al., 2016).

Based on the evidence in neurochemistry and the work of other scholars, the FET views EMP as a trait of behavioural orientation rather than an emotionality trait as it describes preferential behavioural orientation and perceptual sensitivity to other people’s motivation and states. As summarised here, EMP is most likely associated with higher activity OXT, in comparison to VSP and testosterone, with the direct involvement of noradrenaline that likely helps to maintain attention to changeable social aspects of situations.

### Sex Differences in Sensation Seeking and Empathy Might Be a Reflection of Species’ Systemic Factors: Evolutionary Theory of Sex

The analysis of the functionality of neurochemical systems can also benefit from an evolutionary perspective. Sex differences in SS and EMP might be a reflection of species’ self-regulatory systemic factors as outlined in the ETS developed in the 1960s by the mathematical biologist Geodakyan (see Trofimova, 2015 for review). Geodakyan conducted an analysis of sex differences in dispersion patterns, mutation rates, phenotypic and genotypic diversity, birth rates, mortality rates and, susceptibility to new diseases in various species and noted higher variability, higher mortality rates, and higher mutation rate in males in comparison to female phenotypes (Geodakyan, 2000; Trofimova, 2015). In his ETS, he suggested that the benefits of sexual reproduction as a method of reproduction won over other methods because sexual dimorphism allows a species to have two functional partitions:

1) Variational (male) partition for trials and errors for various genetic changes, including parasitic and cooperative co-existence, for possible inter-species co-evolution and expansion of ecological niches (in Geodakyan terms, species use males as “experimental animals of evolution”);
2) Conservational (female) partition to maintain the beneficial features of the species. If, during trial-and-error exploration, a species “experiments” with only a part of the population rather than with the whole aggregation, then another part of the species can continue preserving characteristics that were proven to be beneficial. Studies comparing the genetic transfer in monozygotic male and female twins confirmed this trend.

The ETS, therefore, explains the paradox of sexual reproduction by systemic tendencies for species’ self-regulation. Trofimova pointed to the correspondence of sex differences in verbal-affiliative and exploratory abilities and disabilities with the ETS theory (Trofimova, 2015). Indeed, there is a well-documented superiority in risk- and sensation seeking, physical abilities, higher rates of psychopathy, dyslexia, autism, higher birth and accidental death rates is a reflection of the systemic variational function of the male sex. In contrast, there is female superiority in verbal abilities, lawfulness, socialisation, empathy, and agreeableness, presented as a reflection of the systemic conservational function of the female sex. Figure 1 highlights the sex differences in temperament traits of young males and females, and Figure 3 summarises the interplay between gonadal and social hormones leading to these differences. As could be seen from Figure 3, oxytocin and testosterone indeed act in opposite directions on VSP and the HPA axis (especially adrenaline). OXT pairs its action with estrogen and (not discussed here) serotonin, suppressing VSP, whereas testosterone and KOR activate VSP, which, in turn, activates the HPA axis and NE release (Zhao et al., 1988; Bernstein et al., 1996; Bodnar, 2018). Gonadal hormones, therefore, contribute to sex differences in social perception and self-image, as well as HPA axis arousal (especially the dynamic of adrenaline).

From this perspective, psychological sex differences in communicative and exploratory dis/abilities might not be just an accidental result of sexual selection or labour distribution in early humans. It might be a reflection of a global functional differentiation tendency within a species to expand its phenotypic diversity and at the same time to conserve beneficial features in the species’ behaviour (see Trofimova, 2015 for review).

In summary, the interactions between OR, hormonal and brain neurotransmitter systems in deriving a “emotional verdict” don’t mean that these systems are solely involved in the regulation of emotionality. Similarly, behavioural regulation (including orientation aspects) provided by brain neurotransmitters goes beyond emotional regulation, and, therefore, all of these systems should not be blended into one pot of “emotionality biomarkers.” Heart rate also changes during the intense emotional experience, but we don’t consider the cardiovascular system as a biomarker of emotionality. By analogy, the contribution of vision and visual processing is crucial in behavioural regulation, and it interacts with all other sensory modalities (auditory, proprioceptive, etc.). Yet, conceptually we identify these sensory modality systems as distinct. The same separation should be done with emotionality, and there are benefits in the conceptual differentiation between biomarkers of emotionality. For example, the analysis of contributions from these three of neurochemical systems in social emotions under the condition of tryptophan depletion demonstrated a potential for a more careful and transparent presentation of the interactions between these systems, including an explanation of paradoxical “loss of remorse and shame” in highly neurotic people under tryptophan depletion (Kanen et al., 2021).

Space doesn’t permit us to describe all possible scenarios of psychopathology related to the named systems. We included the notes on them throughout the text, illustrated OR-related psychopathology in the Figure 3, and included several useful references in the last column of Table 1. As Figure 3 shows, there is a “passive approval” case that is often goes under a radar of psychiatrists because people with this condition do not complain. Yet, as seen in our own clinical practice, they have limited functionality (not taking care of their meals, appearance, hygiene, social and personal life, employment) and, therefore, this condition should be included in future classifications of psychopathology. We also want to add that in psychiatric settings, clinical studies using a compact temperament test for screening for the 12 FET-associated temperament traits in people with psychiatric disorders showed the benefits of differentiating between traits related to emotional dispositions and non-emotionality aspects of behavioural regulation. This differentiation was in line with differential symptoms of major depression, generalised anxiety, and other psychiatric disorders (Trofimova and Christiansen, 2016; Trofimova and Sulis, 2016a,b, 2018).

**TABLE 1:** Grouped references regarding the two families of neurochemical systems and their links to psychopathology.

Known as	Function in behavioural regulation	Neurochemical systems and references	Psychopathology associated with these systems (and references)
	**Emotional**	**Amplifiers**	
Emotional valence	Approve or not-approve current d.f.	MOR 54, 55, 62, 63, 77, 204	Dysphoria, low moods (Christie, 2008; Leknes, 2008; Bandelow et al., 2010; Nagi and Piñeyro, 2011; Bodnar, 2018) Borderline PD (Yoshioka et al., 1993; Pan, 1998; Christie, 2008; Bandelow et al., 2010)
		Gut microbiota 64–72	Decrease in inflammatory pain (Bishai et al., 2009) Pain suppression comparable to morphine (Cryan and Dinan, 2012; Dinan and Cryan, 2012; Lach et al., 2017; Ren and Lotfipour, 2020)
Perceptual arousal	Search for alternative and/or new d.f.	KOR 55, 76, 78, 80, 101	Chronic anxiety and agitation (Hauser et al., 2005; Reyes et al., 2008; Schwarzer, 2009; Wittmann et al., 2009; Wee and Koob, 2010; Tejeda et al., 2012; Van’t Veer and Carlezon, 2013; Bodnar, 2018) Indifference, deflation, depression (Schwarzer, 2009; Miller et al., 2018) Ethanol-seeking behaviour (Wee and Koob, 2010; Tejeda et al., 2012) Drug-seeking/addictions (Bruchas et al., 2010; Schindler et al., 2010; Wee and Koob, 2010) Hallucinations and malaise (Schwarzer, 2009; Tejeda et al., 2012) Depersonalisation, thought disorder (Hauser et al., 2005; Tejeda et al., 2012; Van’t Veer and Carlezon, 2013)
		Gut microbiota 68, 91–98 Cytokines 96–98	Anxiety symptoms (Morais et al., 2021) HPA arousal (Haddad et al., 2002; Dunn et al., 2003; Vakharia and Hinson, 2005; Dinan and Cryan, 2012; de Weerth, 2017; Farzi et al., 2018; Misiak et al., 2020; Morais et al., 2021; Salvador et al., 2021) HPA arousal (Haddad et al., 2002; Dunn et al., 2003; Vakharia and Hinson, 2005; Dinan and Cryan, 2012; de Weerth, 2017; Farzi et al., 2018; Misiak et al., 2020; Morais et al., 2021; Salvador et al., 2021)
	**Orientational**	**Aspects**	
Sensation Seeking	Orientation to normalise HPA arousal	(lower) Cortisol 114, 120, 121, 128, 130 Adrenaline 7, 8, 108	Risk-seeking, substance abuse (Zuckerman, 1994, 2007; Hinnant et al., 2016) Social aggression (Montoya et al., 2012; Ponzi et al., 2016; Knight et al., 2017), in combination of high testosterone and low cortisol (Popma et al., 2007; Terburg et al., 2009; Grotzinger et al., 2018) Sympathetic under-arousal (Zuckerman, 1994, 2007) leading to sensation seeking
		Testosterone 105, 114–115, 118, 127, 132	Proneness for violence (Wilson and Daly, 1985)
Empathy	Orientation to others; perception of the other’s presence and motivation	Oxytocin 154, 156, 159–172, 175, 180, 182–186, 196 Vasopressin	Autism (Dinsdale et al., 2013; Kanat et al., 2014; Crespi et al., 2016; Grinevich and Neumann, 2021) Bipolar disorder (Dinsdale and Crespi, 2017) Anxious attachment (Bartz et al., 2015), dependence (Shalvi and De Dreu, 2014) Avoidance behaviour (Bartz et al., 2015) Out-group protective aggression (De Dreu et al., 2016; Daughters et al., 2017) Social psychosis and schizophrenia (Dinsdale et al., 2013; Grinevich and Neumann, 2021) Decrease in fairness with promotion of self-serving dishonesty (Radke and de Bruijn, 2012; Shalvi and De Dreu, 2014; De Dreu et al., 2016; Daughters et al., 2017)
		169, 171, 174, 183, 185, 196	Protective aggression (Heinrichs and Domes, 2008; Kanat et al., 2014)
		Testosterone 155, 194, 198–200 (low)	Self-centred tendency (Crespi, 2016; Crespi et al., 2016) Schizotypy (low oxytocin, high testosterone) (Dinsdale et al., 2013; Crespi, 2016) Social aggression (Montoya et al., 2012; Ponzi et al., 2016; Knight et al., 2017), in combination of high testosterone and low cortisol (Popma et al., 2007; Terburg et al., 2009; Grotzinger et al., 2018)

*Psychopathology can be associated with extreme excesses or extreme deficiency of receptor density and/or supplies in binding chemical agents, bringing a spectrum of possible clinical scenarios. MOR/KOR, mu/kappa opioid receptors; d.f., degrees of freedom in behaviour.*

## Conclusion

This review is an attempt to disentangle the neurochemical systems implicated in emotionality using the FC approach and the FET framework. The neurochemical FET framework summarises the most consistent and experimentally verified findings reported earlier, classified into three emotionality and nine non-emotionality-related universal aspects of action construction. This review highlighted the benefits of further distinguishing between systems (1) regulating emotional dispositions experienced before the events, (2) regulating reactivity to fast-changing immediate events, and (3) preparation of “eventual” behaviour related to probabilistic aspects of events.

Emotionality traits are presented here as emotional dispositions based on OR systems. Two out of three emotional dispositions included in the FET framework were discussed here:

- MOR-based emotional valence, inducing a dispositional sense of approval or disapproval with current and projected events; as noted, MOR action is entangled with the action of immune system suppressing cytokines and pain.
- KOR-based perceptual and behavioural alertness, inducing a disposition to search for alternative d.f. in behaviour; as noted, KOR action is also entangled with the action of the HPA and immune systems, increasing alertness in response to cytokines.

Here we highlighted the evidence that even though hormonal and OR systems are traditionally seen as all regulating emotionality, they differ in their functions in behaviour. The differences in their functionality could be used to disentangle emotional dispositions based on the state of the body from non-emotionality aspects of behavioural regulation related to the state of surrounding events.

We proposed to consider dispositional emotionality associated with OR systems as emotionality in a true sense. In contrast, hormones could be seen as neurochemical biomarkers of non-emotional aspects of behavioural regulation related to the construction of behaviour in fast-changing and current situations. As examples of hormonal regulation of orientation-related temperament traits, the paper reviewed neurochemical systems of SS and EMP and pointed to the need to differentiate them from emotionality-related traits such as Neuroticism and dispositional Satisfaction. The literature reviewed here presents SS as based on (higher) testosterone, (fluctuating) adrenaline and (low) cortisol systems, and EMP as based on (higher) oxytocin, reciprocally coupled with vasopressin and (lower) testosterone. Due to the involvement of gonadal hormones, there are sex and age differences in these traits that could be explained by evolutionary theory.

Current debates about how many basic emotions exist and how they can be partitioned and classified could benefit from references to neurochemical biomarkers, as described above. The functional and anatomic segregations within these biomarkers clearly suggest a differentiation between a dispositional type of emotionality (and within it – three OR-based subsystems of dispositional emotionality, two of which were discussed in this review) and non-emotionality aspects of behavioural regulation. Non-emotionality aspects relate to social, physical and intellectual functioning to fast-changing events vs. implicit, distant, and not immediate (probabilistic) features of reality. In this sense, there are a number of non-emotionality aspects that could create a diversity of emotional experiences when blended with emotional dispositions (“social emotions,” “emotional intelligence,” “stress,” etc.). This review highlighted the benefits of differentiating between emotional dispositions and non-emotionality aspects for the development of neuroscience-based models of emotionality.

The limitations of this review relate to inability to grasp all the aspects neurochemical regulation of emotionality. For example, more could be said about the role of glutamate and GABA neurotransmitters, or the recently uncovered roles of BDNF and CREB in emotionality. The work in a conceptual differentiation between neurochemical systems regulating emotionality aspects of behaviour is still a work in progress, and the FET is one of many frameworks that could be used for such differentiation. We view future directions in multi-disciplinary integrations between neurochemistry, gut psychiatry and temperament research, as well as the inclusion of taxonomies of behavioural contexts in classifications related to emotional regulation.

## Author Contributions

IT designed the concept of the manuscript, wrote the drafts, designed and produced the figures, did the literature search, and formatted the references. AG contributed to the drafts, did the literature search, edited the figures, and formatted the references. Both authors contributed to the article and approved the submitted version.

## Conflict of Interest

The authors declare that the research was conducted in the absence of any commercial or financial relationships that could be construed as a potential conflict of interest.

## Publisher’s Note

All claims expressed in this article are solely those of the authors and do not necessarily represent those of their affiliated organizations, or those of the publisher, the editors and the reviewers. Any product that may be evaluated in this article, or claim that may be made by its manufacturer, is not guaranteed or endorsed by the publisher.

## Abbreviations

NTs: – neurotransmitters: 5-HT, 5-hydroxytryptamine (serotonin)
Ach: acetylcholine
NE: noradrenaline
DA: dopamine
NP: neuropeptides
Glu: glutamate
GG: GABA and Glu
A: adrenaline
GPCRs: G protein-coupled receptors
KOR, MOR and DOR: kappa-,mu-, and delta-opioid receptors correspondingly. Brain structures: PFC, (dorsal, medial) prefrontal cortex
OFC: orbito-frontal cortex
HT: hypothalamus
HC: hippocampus
LC: locus coeruleus
(dL, V)TA: (dorsolateral, ventral) tegmental area
cAM: (central)amygdala
NAc: nucleus accumbens
HPA: hypothalamic–pituitary–adrenal axis
HPG: hypothalamic–pituitary–gonadal axis
ACTH: adrenocorticotropic hormone
CRH: corticotropin-releasing factor
FET: Functional Ensemble of Temperament framework to classify neuro-chemically based behavioural aspects
FC: functional constructivism
CBPs: consistent behavioural patterns.
